# Occurrence and antimicrobial susceptibility patterns of *Escherichia coli* and *Salmonella* species in raw beef from abattoir and retailer shops in Nairobi, Kenya

**DOI:** 10.3389/fmicb.2026.1808597

**Published:** 2026-06-15

**Authors:** Caroline C. Kasiwai, Joshua M. Njiru, Maina J. Wagacha, Evans N. Nyaboga

**Affiliations:** 1 Department of Biology, University of Nairobi, Nairobi, Kenya; 2 Department of Testing, Kenya Bureau of Standards, Nairobi, Kenya; 3 Department of Research and Development, Kenya Bureau of Standards, Nairobi, Kenya; 4 Department of Biochemistry, University of Nairobi, Nairobi, Kenya

**Keywords:** abattoir and informal markets, antimicrobial resistance, *Escherichia coli*, multidrug resistance, raw beef, *Salmonella* spp.

## Abstract

Microbiological safety of beef meat is critical for public health, particularly due to the rising concern of antimicrobial resistance (AM) in foodborne pathogens including *Escherichia coli* and *Salmonella* spp. The aim of this study was to determine the occurrence and prevalence of *E. coli* and *Salmonella* spp. in raw beef meat from abattoir and butcher shops and assess the antimicrobial susceptibility patterns of the bacterial isolates. A total of 106 beef cuts were sampled from abattoirs and randomly selected butcheries from Dagoretti sub-County, Nairobi. Antibiotic susceptibility was determined in fifty *E. coli* isolates and seventeen presumptive *Salmonella* spp. isolates using the disk diffusion method. Antibiotic resistant genes were confirmed by polymerase chain reaction amplification and multidrug-resistant (MDR) *E. coli* were identified by 16S rRNA sequences. The *E. coli* and *Salmonella* isolates were 100% (*n* = 50 and *n* = 17, respectively) resistant to Ampicillin/cloxacillin, 62% (*n* = 31) and 82% (*n* = 14) resistant to Tetracycline, 42% (*n* = 21) and 47% (*n* = 8) resistant to Co-trimoxozole, 44% (*n* = 22) and 58.8% (*n* = 10) resistant to Erythromycin, 20% (*n* = 10) and 23.5% (*n* = 4) resistant to Nalidixic acid, 10% (*n* = 5) and 23.5% (*n* = 4) resistant to Chloramphenicol and 12% (*n* = 6) and 23.5% (*n* = 4) resistant to Kanamycin, respectively. *Escherichia coli* and *Salmonella* isolated from beef had high antimicrobial resistance to commonly used antibiotics. Polymerase chain amplification revealed that all the tesed *E. coli* and *Salmonella* isolates carried at least two of the screened antibiotic-resistant genes, with *bla*_*TEM*_ and *bla*_*CMY*–2_ present in all of them. Phylogenetic analysis showed that the eight MDR *E. coli* isolates were similar to *E. coli* strains isolated in other countries which were pathogenic *E. coli*. The study highlights significant antimicrobial resistance and the presence of antibiotic resistant genes in *E. coli* and *Salmonella* spp. isolates from raw beef meat. Overall, the outcome of this study regarding antimicrobial-resistant *E. coli* and *Salmonella* in beef meat samples presents a significant risk to human and animal health.

## 1 Introduction

Microbiological safety of beef meat is a major public health concern worldwide, as it represents a potential vector for transmitting resistant pathogenic bacteria to humans (Chepkemei et al., 2022). According to the World Health Organization (WHO), consumption of contaminated food causes 600 million illnesses and 420,000 deaths annually (World Health Organization [WHO], 2024). *Escherichia coli* and *Salmonella* species are the most common bacteria that cause foodborne illnesses affecting millions of people annually and sometimes causing severe and fatal outcomes (Lahiri et al., 2010; World Health Organization [WHO], 2015). Pathogenic *E. coli* and *Salmonella* spp. inhabit the gastrointestinal tract of domestic and wild animals especially those raised for human consumption (Zhao et al., 2001). Raw or undercooked beef meat is a significant source of *E. coli* and *Salmonella* spp. infections to humans and contamination with these pathogens occur at different stages from slaughter to retail (Zhao et al., 2001; Getenesh et al., 2020; Wambui et al., 2017; Abebe et al., 2020). For instance, studies on butcher shops in Kenya concluded that butchery operators did not adhere to the required sanitation and hygiene practices which could lead to an increase in microbial contamination and occurrence of pathogenic microorganisms. In the same study, *Listeria* spp., *Pseudomonas* spp., *E. coli* and other microorganisms were isolated from beef meat sold in Nairobi and Isiolo Counties (Kunyanga et al., 2021; Chepkemei et al., 2022; Chepkemoi et al., 2015). In Kenya, the traditional habit of consuming raw or undercooked beef creates an ideal situation for the spread of *E. coli* and *Salmonella* spp. (Chepkemei et al., 2022; Dabasso et al., 2022). Therefore, understanding microbiological meat safety is essential for understanding consumer exposure to *E. coli* and *Salmonella* spp. and the associated risk of antimicrobial resistance (AMR).

Bacterial pathogens including *E. coli* and *Salmonella* spp., contaminating food have developed resistance to antibiotics that were once effective (O’Neil, 2016). The misuse and overuse of antibiotics in human health and animal production have contributed to the emergence of multidrug-resistant (MDR) and extensively drug-resistant (XDR) *E. coli* and *Salmonella* spp. strains, complicating treatment options and increasing the risk of disease outcomes (Velazquez-Meza et al., 2022; Elshebrawy et al., 2021; Magiorakos et al., 2012). Antimicrobial resistance is reported to be high and rising in *E. coli* and *Salmonella* spp., posing a significant public health threat (Mayrhofer et al., 2004; Hamilton et al., 2024; Musuka et al., 2025; World Health Organization [WHO], 2021). A study in Ghana has shown that *E. coli* isolates have increasingly become resistant to commonly used antibiotics such as Erythromycin, Tetracycline and Ampicillin with a resistance rate of 85, 73, and 71%, respectively and a 68% multidrug resistance rate (Adzitey et al., 2020). *Salmonella* spp. isolates have also been shown to be resistant to commonly used antibiotics including Ampicillin, Amoxicillin, Tetracycline and Gentamycin (Bawa et al., 2020). Since antimicrobial resistant bacterial pathogens can be transferred to humans from animals through the food chain (Florez-Cuadrado et al., 2018), foods of animal origin such as beef meat may serve as reservoirs and conduits of AMR (Economou and Gousia, 2015; Zhao et al., 2012). Therefore, there is need to understand the maintenance and dissemination of AMR along the food supply chain (Zawack et al., 2016).

Although there has been substantial research on food borne pathogens and antimicrobial resistance in pigs and poultry, there is lack of targeted research specifically on the prevalence of *E. coli* and *Salmonella* spp. in beef meat from abattoirs and retail butcher shops in Nairobi and their antimicrobial resistance patterns. Despite the growing urgency to address AMR, there is a lack of baseline data on the prevalence and resistance profiles of foodborne pathogens, particularly *E. coli* and *Salmonella* spp., in beef meat sold in abattoir and informal retail markets in Kenya. In addition, limited studies have reported the detection of antibiotic resistance genes in *E. coli* and *Salmonella* spp., isolated from raw meat in Kenya. The objective of this study was therefore to determine the prevalence of pathogenic *E. coli* and *Salmonella* spp. in raw beef sold in Dagoretti sub-County, Nairobi and to analyze their antimicrobial susceptibility patterns to inform public health strategies and improve food safety practices. The findings from this study could provide important data for development of effective public health strategies tailored to reduce contamination and antimicrobial resistance.

## 2 Materials and methods

### 2.1 Description of the study area

This study was carried out in Dagoretti North and South sub-Counties, Nairobi County (Supplementary Figure S1). Dagoretti North and South sub-Counties are located in the Western part of Nairobi and are among the 17 sub-counties of Nairobi County. Dagoretti South has four wards namely Mutuini, Ngando, Riruta and Waithaka; while Dagoretti North has five wards namely Kilimani, Kawangware, Gatina, Kileleshwa and Kabiro. The Dagoretti livestock market is in two sub-Counties (Dagoretti North and South) and is located at the border of Nairobi and Kiambu Counties. It is the largest livestock market also referred to as Ndonyo which means “the marketplace” in Kikuyu language and it serves the residence of Nairobi, Kiambu and Kajiado Counties (Wafula and Wasonga, 2021). There are about 16 slaughterhouses in the Dagoretti livestock market where approximately 1,200 1,600 cattle are slaughtered per week (Alarcon et al., 2017), which primarily serve wholesale and retail purposes. These abattoirs serve the residents of Dagoretti and its environs including Waiyaki way, Karen, Ngong road, Kikuyu and Langata road. While most of the animals slaughtered in Dagoretti are sourced from nearby regions, Narok County serves as a significant source of livestock for the abattoir (Alarcon et al., 2017).

### 2.2 Determination of sample size and sampling

The formula by Fisher and Yates (1990) was used to calculate the sample size. In a study conducted by FAO/WHO on improving food safety in meat value chain in Kenya, non-typhoidal *Salmonella* and *Shigella* were isolated at an average of 7% from children hospitalized with diarrhea in rural, city and under-privileged areas in Kenya. Therefore, with the use of a prevalence rate (P) of 7%, a standard error from the mean (Z) of 1.96 and an absolute degree of precision (d) of 5%, 106 samples were collected and analyzed in this study.

Beef cut samples were obtained randomly from the abattoirs and retail butcheries within Dagoretti South and North sub-counties (Table 1). The sampling locations were stratified into slaughterhouses (five abattoirs) and butcheries in Dagoretti South and Dagoretti North. Simple random sampling design was used to obtain samples from the butcheries. Sample collection was carried out as described in ISO 17604:2015 (International Organization for Standardization, 2015), labeled and immediately transported to the laboratory using a cooler box containing frozen freezer blocks. Beef samples ranging from 100 to 200 g were purchased from the butcheries and slaughterhouses. The procedure involved the normal handling, cutting and packaging of beef by the butcher using the tools and equipment typically used during the routine sale of meat. This approach was intended to reflect typical market practices in order to determine the safety of meat offered for public consumption. After purchase, the samples were transferred to sterile stomacher bags using sterile forceps which were sterilized using cotton wool dipped in 70% ethanol. The samples were kept in a cooler box maintained at a temperature of between 1 and 8°C before transportation to the laboratory and were analyzed immediately after receipt. Samples which could not be analyzed immediately were stored in a refrigerator at 4 ± 2°C for a maximum of 24 h at the Kenya Bureau of Standards (KBS) Microbiology Laboratory before analysis.

**TABLE 1:** The number of beef samples and prevalence of *E. coli* and *Salmonella* spp. in beef from abattoirs and retail butcheries in different wards in Dagoretti South and North Sub-counties, Nairobi.

Sub-county	Type of facility	Ward	No. of samples	Samples positive for *E. coli*	Samples presumptive for *Salmonella* spp.
Dagoretti North	Abattoir	Kawangware	10	7 (70%)	5 (50%)
	Retail butchery	Gatina	7	3 (43%)	1 (14%)
		Kabiro	13	13 (100%)	0 (0%)
		Kilimani/Kileleshwa	9	3 (33%)	2 (22%)
		Kawangware	9	7 (78%)	2 (22%)
Dagoretti South	Retail butchery	Waithaka	10	2 (20%)	2 (20%)
		Riruta	10	9 (90%)	3 (30%)
		Mutu-ini	9	8 (89%)	2 (22%)
		Ngando	13	12 (92%)	3 (23%)
		Uthiru	16	10 (63%)	3 (19%)
Total			106	74 (69.8%)	23 (21.7%)

### 2.3 Isolation and identification of *E. coli*

Isolation and identification of *E. coli* was done according to the International Organization of Standardization (ISO) method ISO 16649-2:2001 (International Organization for Standardization, 2001) with minimal modifications. The samples were prepared for testing according to specific rules for the preparation of meat and meat products as per ISO 6887-2:2017 (International Organization for Standardization, 2017b). Sterile forceps and scissors were used to cut the beef into small pieces. Approximately 10 g sub-sample was weighed into a sterile stomacher bag, and 90 mL of buffered peptone water (BPW, Oxoid Ltd., Basingstoke, United Kingdom) was added and the bag was put into a stomacher. The samples were homogenized at 8 strokes per s for 2 min. After the homogenization, each bag was closed and incubated at 37°C for 24 h. An aliquot of the overnight broth culture was then streaked onto Tryptone Bile X-Glucuronide (TBX) agar plates (Biolife, Milan, Italy) by using a sterile 10-μL loop and incubated at 42°C for 24 h. The isolates that presented typical E. coli morphology were selected for subsequent analysis The typical colony forming units (CFU) of glucuronidase positive *E. coli* presenting as blue green colonies in each dish were counted.

### 2.4 Isolation and identification of *Salmonella*

#### 2.4.1 Pre-enrichment in non-selective medium

*Salmonella* spp. was isolated according to ISO 6579-1:2017, horizontal methods for the detection, enumeration and serotyping of *Salmonella* spp. (International Organization for Standardization, 2017a). Sterile forceps and scissors were used to cut the beef meat into small pieces (approximately 2 g). Approximately 25 g sub-sample was weighed into a sterile stomacher bag, and 225 mL of buffered peptone water (BPW) was added. The mixture was homogenized by blending using a stomacher blender. This mixture formed the initial suspension (pre-enriched samples). The pre-enriched samples in BPW were incubated at 37°C for 18 ± 2 h.

#### 2.4.2 Enrichment in selective broth media

For the selective enrichment stage, 0.1 mL of the culture obtained from pre-enrichment was transferred into a tube containing 10 mL of the Rappaport Vassiliadis soya peptone broth (RVS broth). The RVS broth was incubated at 42 °C for 24 h (International Organization for Standardization, 2017a).

#### 2.4.3 Selective plating out and isolation

The cultures obtained from RVS were inoculated using a 10 μL loop onto the surface of Xylose Lysine Deoxycholate (XLD) agar plate to obtain well isolated colonies. The XLD plates were inverted during incubation at 37°C for 24 h. The typical colonies of *Salmonella* spp. on XLD agar were observed as a black center and a lightly transparent zone of reddish color due to the color change of the indicator. Presumptive/typical *Salmonella* spp. colonies from XLD agar were sub-cultured on nutrient agar and incubated at 37°C for 24 h to obtain pure colonies. Biochemical confirmation of typical *Salmonella* spp. was carried out using triple sugar iron agar (TSI) and Microbact 24 E identification kit (International Organization for Standardization, 2017b). *Salmonella typhimurium* ATCC 13311 reference strain was used as positive control for the isolation of *Salmonella*.

### 2.5 Biochemical characterization of suspected isolates of *Salmonella* species

The biochemical characterization of the suspected *Salmonella* spp. was performed by different biochemical tests and the results were interpreted according to the guidelines of the International Organisation for Standardization (International Organization for Standardization, 2017a). The pure colonies cultured on NA (Oxoid Ltd., Basingstoke, United Kingdom) were used for biochemical test, Triple Sugar Iron (TSI) test.

Oxidase test was done using the Oxoid oxidase strips product code MB0266 (ThermoFisher Scientific, United Kingdom). The isolates were tested with Microbact 24E system (Oxoid Ltd., Basingstoke, United Kingdom) and the results interpreted according to the manufacturer’s instructions. Biochemical tests available on Microbact 24E system were: Lysine decarboxylase (LSY), Ornithine decarboxylase (ORN), hydrogen sulfide (H_2_S), glucose (GLU), Acid from manittol (MAN), Acid from xylose (XLY), β-Galactosidase (ONPG), Indole production (IND), urea hydrolysis (URE), Voges-Proskauer reaction (VP), Citrate utilization (CIT), Tryptophan deaminase (TDA), Gelatine liquefacaton (GEL), Malonate utilization (MAL), inositol (INO), sorbitol (SOR), rhamnose (RHA), sucrose (SUC), lactose, (LAC) arabinose (ARA), Adonitol (ADO), raffinose (RAF), salicin (SAL) and arginine (ARG) used for the identification of Enterobacteriaceae and common miscellaneous Gram-negative bacilli (MGNB). The reactions were evaluated as positive or negative based on the color chart provided by the manufacturer. Serological confirmation of *Salmonella* spp. was performed for all the isolates that were confirmed to be positive using the microbact 24 E kit. The serological analysis was done based on slide agglutination test using Poly “O” and “H” antisera (Remel, United Kingdom).

### 2.6 Antimicrobial susceptibility testing of *E. coli* and Salmonella isolates

The confirmed pathogenic isolates of *E. coli* and *Salmonella* spp. were subjected to antimicrobial susceptibility testing. Kirby-Bauer disk diffusion susceptibility test protocol was used (Mumbo et al., 2023). Mueller-Hinton agar was used for the disk diffusion method. The organisms to be tested were purified by culturing on nutrient agar at 37°C for 24 h. In a biosafety cabinet, at least five colonies from the nutrient agar plate (overnight culture) were touched using a sterile inoculating loop, suspended into 2 mL sterile saline and then vortexed. The turbidity was adjusted to 0.5 McFarland standard and the suspension was used within 15 min of preparation. A sterile swab was dipped into the suspension and used to inoculate the dry surface of Mueller-Hinton agar by streaking the swab three times over the entire agar surface, the plate was rotated approximately 60° each time to ensure even distribution of the inoculum. The plate was then allowed to dry at room temperature (23 ± 2°C) for 3–5 min. Antibiotic impregnated disks (Seven disks in a 150 mm Petri dish) were dispensed onto the surface of inoculated Mueller-Hinton agar then incubated at 35°C for 16–18 h. *Salmonella* spp. and *E. coli* isolates were tested for susceptibility to seven selected commonly used antibiotics from seven classes of antibiotics namely Penicillin (Ampicillin/Cloxacillin [AX_10_, 10 μg]), Macrolide (Erythromycin [E_15_, 15 μg]), Quinoline (Nalidixic acid [NA_30_, 30 μg]), Aminoglycoside (Kanamycin [K_30_, 30 μg]), Tetracycline (Tetracycline [TE_30_, 30 μg]), Folate-pathway antagonist (Co-trimoxazole [COT_25_, 25 μg]) and Phenolic derivatives (Chloramphenicol [C_50_, 50 μg]). Appropriate quality control (QC) strains (ATCC strain) of *E. coli* ATCC 25922 and *S. typhimurium* ATCC13311 as extra checks on test parameters were used. The results of antimicrobial susceptibility testing were interpreted according to guidelines set by the Clinical Laboratory Standards Institute (Clinical and Laboratory Standards Institute, 2024). Multidrug resistance was defined as resistance to at least three classes of antibiotics used in the study. The multiple antimicrobial resistance (MAR) index was determined as described previously (Krumperman, 1983).

### 2.7 Molecular identification and characterization of *Escherichia coli* and *Salmonella* species

#### 2.7.1 Detection of antimicrobial resistance genes associated with pathogenic bacteria

Resistance genes (Supplementary Table S1) that encode resistance to the different classes of antibiotics were detected as described by Mumbo et al. (2023). Strains *E. coli* MAK-26 and *S. typhimurium* MAK-22 were used as positive controls for PCR reactions for detection of antibiotic resistance genes in MDR bacteria isolates (Mumbo et al., 2023).

#### 2.7.2 Molecular identification of multidrug resistant *E. coli* isolates

Isolates of *E. coli* that showed multidrug resistance were identified based on molecular analysis using 16S rRNA primer sequence as described by Mumbo et al. (2023). The 16S rRNA primer pair (forward B27F 5’-GAGTTTGATCCTGGCTCA-3’and reverse 1492R 5’-TACGGCTACCTTGTTACGACTT-3’) amplified amplicons of approximately 1,497 bp.

##### 2.7.2.1 Genomic DNA extraction

Total genomic deoxyribonucleic acid (DNA) extraction was done as described by Mumbo et al. (2023). Single purified colonies of each of the *E. coli* and *Salmonella* spp. isolates were enriched in 10 mL nutrient broth and incubated at 37°C for 24 h. Approximately 1 mL of the microbial enrichment was transferred to a reaction tube and the cell suspension centrifuged for 10 min at 10,000 × g after which the supernatant was discarded. The sediment was resuspended in 1 ml physiological saline (wash buffer) and the cell suspension centrifuged for 10 min at 10,000 × g after which the supernatant was discarded. The sediment was re-suspended in 250 μL of water where thermal digestion was carried out by heating for 20 min at 95°C for *Salmonella* spp. and for 15 min at 100°C for *E. coli* isolates. Afterward, the centrifuge tubes were transferred to an ice-bath to cool quickly. The lysate was mixed vigorously using a vortex mixer then centrifuged at 25°C for 3 min at 10,000 × g for *Salmonella* spp. and 10 s at 10,000 × g for *E. coli*. The supernatant was transferred to a new microliter reaction tube. The quality of the extracted DNA was confirmed using Nanodrop spectrophotometer (JENWAY™ Genova Nano 83070-02, Leicestershire, United Kingdom) as described in Addgene protocol.^1^ The spectrophotometer was calibrated using the same water the DNA was resuspended in but without any DNA. Then 1 μL of the extracted DNA was placed in the Nanodrop pedestal and the lid closed. The concentration and purity of DNA was measured and recorded.

##### 2.7.2.2 Polymerase chain reaction amplification and agarose gel electrophoresis

Polymerase chain reaction (PCR) amplifications were carried as described by Mumbo et al. (2023). The total PCR reaction volume was 25 μL containing 2.5 μL of 10 × PCR buffer, 0.5 μL of 10 mM dNTP mix, 1.2 μL of 25 mM MgCl_2_, 2.5 μL each of 10 μm forward and reverse primer, 0.125 μL Taq polymerase (5 U/μL), 2 μL of template DNA and topped up with approximately 15 μL of distilled water. The PCR cycling program was initial denaturation for 15 min at 95 °C, denaturation for 0.5–1 min (depending on the primer used) at 95 °C, annealing 0.5–1 min 50–68 °C (depending on the primer used), extension for 1 min at 72 °C and final extension for 10 min at 72 °C. The number of cycles for denaturation, annealing and extension were thirty five. Amplified PCR products (5 μL) were subjected to agarose gel electrophoresis as per Addgene protocol. Agarose gel 1% (w/v) stained with Gel Red in 1 × TAE buffer was used to confirm the presence of amplified PCR products. The electrophoresis was set at 80 V until the bromophenol blue dye line was approximately 75% of the way down the gel. Power was turned off and the gels were visualized under ultraviolet light trans illuminator-Quantum (ST4 1100-26MX Biotech, Marne-la-Vallee, France).

##### 2.7.2.3 Sequencing, sequence analysis and phylogenetic analysis

The PCR amplicons were purified using QIA-quick kit (Qiagen, Hilden, Germany) and sent to macrogen, Europe for sequencing. Sanger sequencing technique was used to sequence the samples in both the 5’–3’ direction and 3’–5’ direction. In the National Centre for Biotechnology Information (NCBI) website, the Basic Local Alignment Search Tool for nucleotides (BLASTn) was selected, and the cleaned sequences were pasted in the nucleotide blast option with the program selection for highly similar sequences. BLASTn was used to calculate sequence similarity and identify evolutionary relationships between sequences (NCBI).^2^ The percent identity and expected value (E value) were used as parameters to determine whether the alignment was good and if there was a possible biological relationship. The sequences generated by BLAST with E values closer to zero and percent identity of more than 90% were selected (Madden, 2002).

Multiple sequence alignment (MSA) of 16S rRNA sequences obtained was performed using Multiple Sequence Comparison by Log-Expectation (MUSCLE) (Edgar, 2004). The multiple sequence alignment was trimmed using BioEdit sequence alignment editor version 7.7.1 to remove low quality ends and indels (insertions and deletions). The resulting multiple sequence alignment was rendered in ESPript 3 (see text footnote 2) to show the conservation and identify highly variable positions (Robert and Gouet, 2014). The MSA of 16S rRNA sequences was used to construct a phylogenetic tree using MEGA (Molecular Genetics Analysis) version 11 (Tamura et al., 2021). The evolutionary distances were computed based on Kimura 2-parameter (K2P) model using Neighbor-Joining (NJ) and/or Maximum Likelihood (ML) methods. The DNA sequences of 16S rRNA were submitted to the GenBank via the Submission Portal^3^ and assigned accession numbers.

### 2.8 Data management and analysis

A descriptive study design was used and the data that was collected in the study was both quantitative and qualitative. Results were presented by using Tables, bar graphs and histograms. Data analysis was done using excel statistical software. Analysis of variance (ANOVA) was used to determine significant differences of the mean microbial counts of similar pathogens obtained from different sampling locations. Tukey’s test was used to separate the means at 5% significance level (*P* ≤ 0.05).

## 3 Results

### 3.1 Prevalence of *Escherichia coli* and *Salmonella spp.* in beef samples

**FIGURE 1:**
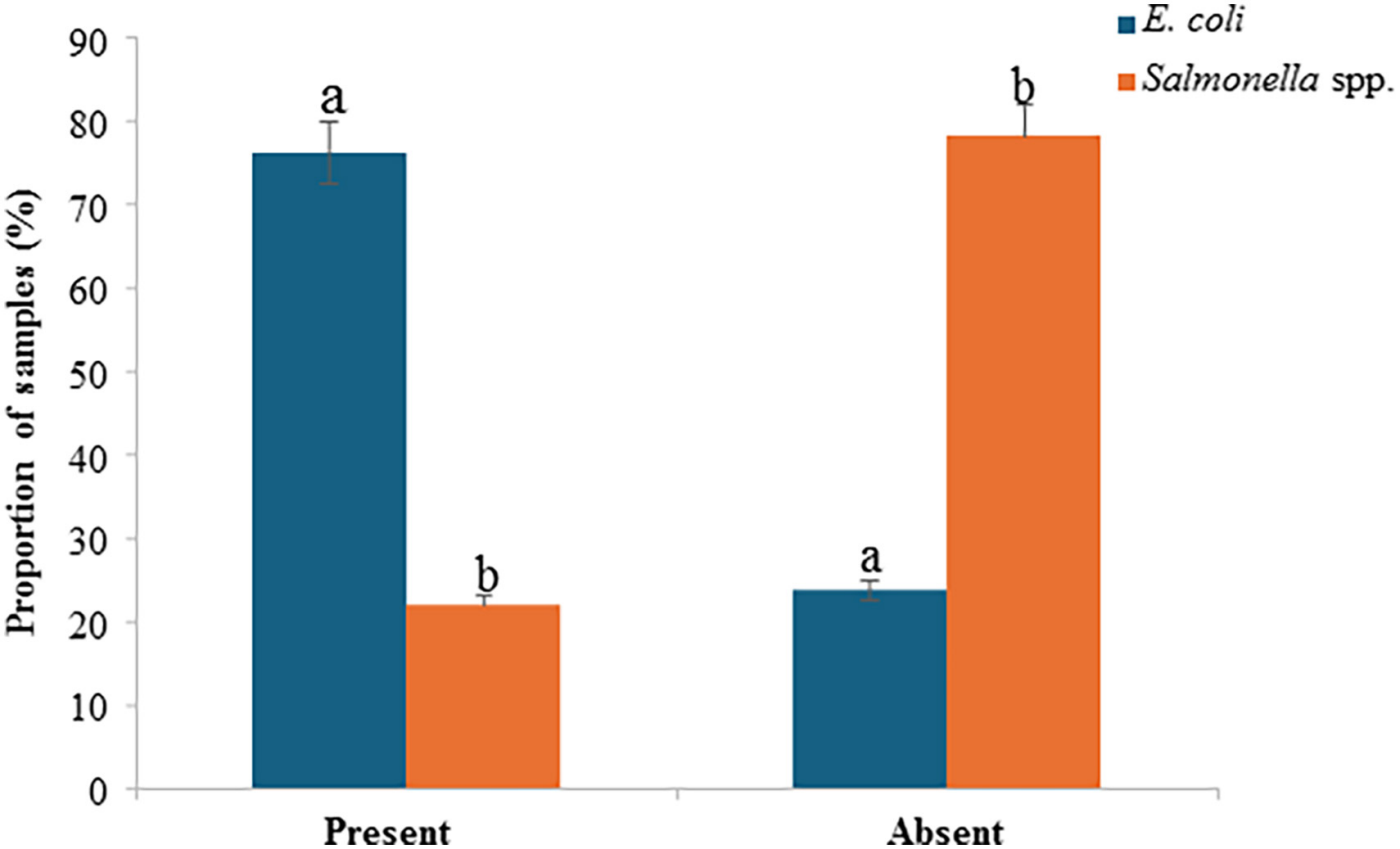
Proportion (%) of *E. coli* and number of presumptive *Salmonella* spp. isolated from 106 beef samples collected from different sampling sites in Dagoretti North and South sub-Counties. The error bars represent standard error of the means. Bars having different lower case letters for presence for *E. coli* and *Salmonella* are significantly different at *p* ≤ 0.05. Bars having different lower case letters for absence of *E. coli* and *Salmonella* are significantly different at *p* ≤ 0.05.

*Escherichia coli* was isolated from 74 (69.8%) beef samples, while presumptive *Salmonella* spp. were isolated from 23 (21.7%) beef samples on XLD agar (Figure 1). However, only six isolates (5.7%) were confirmed as *Salmonella* spp. using Microbact ™ Gram negative 24E identification kit. Only two samples were not contaminated with *E*. *coli* and *Salmonella*. The difference in isolation frequencies between *E. coli* and *Salmonella* spp. was statistically significant (χ^2^ = 28.81, p = 7.986e-08). The population of *E. coli* and *Salmonella* spp. in the different sampling sites is summarized in Table 1.

### 3.2 Characteristics of *Escherichia coli* and *Salmonella* spp. in beef samples

*Escherichia coli* isolates displayed the expected, purple-colored colonies with green metallic sheen on EMB (Levine) agar. *E. coli* isolates displayed the expected blue-green colored colonies on TBX agar (Supplementary Figure S2). The colonies were smooth, circular, 2–3 mm in size and with convex elevation. Isolates of *Salmonella* spp. displayed the expected red colonies with black centers on XLD media, and the colonies were 2–3 mm in diameter. *Salmonella* spp. on TSI displayed a red slant yellow/black butt and gas (Supplementary Figure S3). *Salmonella* spp. is a facultative anaerobe, catalase positive and oxidase negative. The presumptive *Salmonella* spp. isolates were confirmed using Microbat ™ Gram negative identification kit 24E. *Salmonella* spp. was confirmed to be present in six samples. Three confirmed *Salmonella* spp. isolates were recovered from beef samples collected from Ndonyo; two isolates from samples from Kilimani/Kileleshwa; while one isolate was recovered from a sample from Kabiro.

### 3.3 Counts of *Escherichia coli* and *Salmonella* spp. in beef samples

**FIGURE 2:**
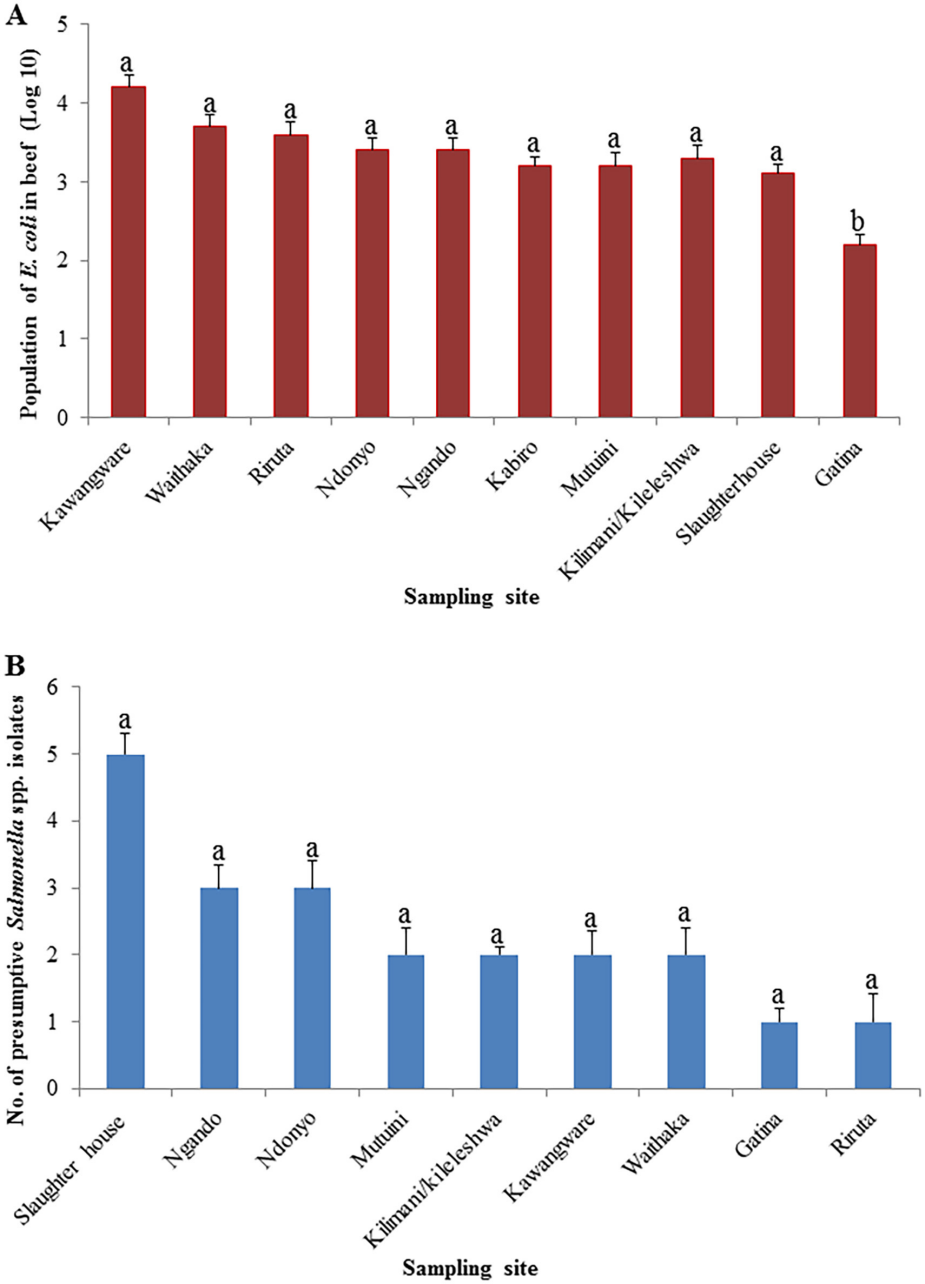
Population of *E. coli* (Log _10_) and presumptive *Salmonella* spp. isolated from 106 beef samples collected from different sampling sites in **(A)** Dagorretti North and **(B)** Dagoretti South sub-Counties. Values are means ± standard errors of triplicate measurements. For Dagoretti North sub-County **(A)**, bars having similar lower case letters are not significantly different at *p* > 0.05. For Dagoretti South sub-County **(B)**, bars having similar lower case letters are not significantly different at p > 0.05.

There was no significant difference in the mean counts of *E. coli* (Log_10_) (*p* = 0.19) and number of presumptive *Salmonella* spp. (*p* = 0.51) isolates from Dagoretti North and South sub-Counties. The average population of *E. coli* isolates from the different sampling sites ranged from Log_10_ 2.2 to Log_10_ 4.2 colony forming units (Figure 2). The difference in the population of *E. coli* for the different sampling sites was statistically significant (*p* = 0.049). A tukey’s honestly significant difference (HSD) *post-hoc* test showed there was significant difference between Gatina and Kawangware (*p* = 0.02) with no significant differences in the other sampling sites (*p* > 0.05).

The presence of presumptive *Salmonella* spp. differed among the sampling sites. Five presumptive *Salmonella* spp. isolates were recovered from beef samples from Dagoretti abattoir; while three presumptive *Salmonella* spp. isolates were recovered from samples from Ngando and Ndonyo. Two presumptive *Salmonella* spp. isolates each were recovered from beef samples from Mutuini, Kilimani/Kileleshwa, Kawangware and Waithaka; one presumptive *Salmonella* spp. isolate was recovered from samples collected from Gatina and Riruta (Figure 2). However, the population of presumptive *Salmonella* spp. was not significantly different (*p* = 0.54) for beef samples from the different sampling sites (Figure 2).

### 3.4 Antimicrobial susceptibility patterns of *Escherichia coli* and *Salmonella* spp.

#### 3.4.1 *Escherichia coli* susceptibility patterns

**FIGURE 3:**
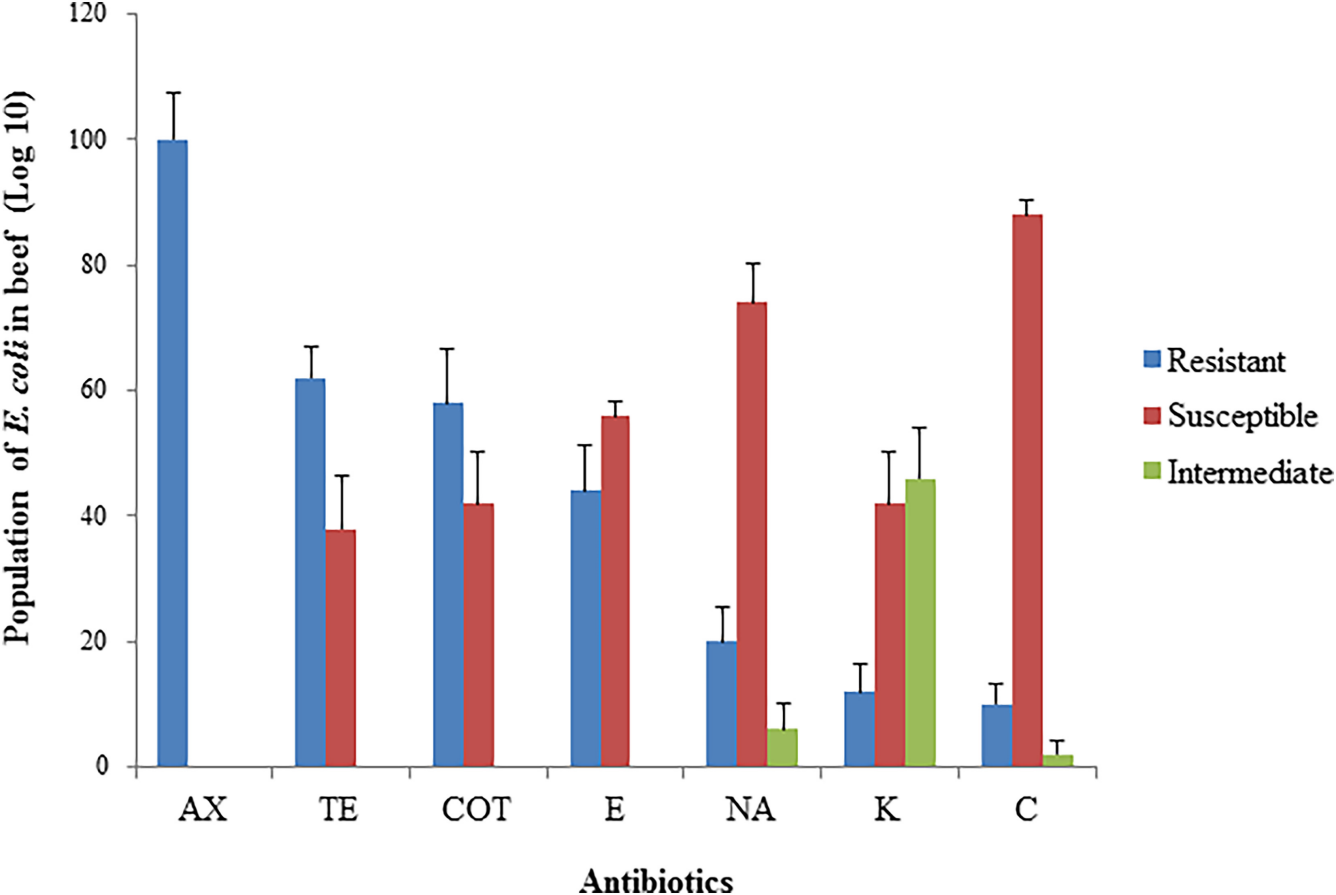
Antibiotic reaction of *E. coli* isolates from different sampling sites in Dagoretti North and South sub-Counties to different antibiotics. The error bars represent standard error of the means. AX, ampicillin/Cloxacillin; E, erythromycin; NA, nalidixic acid; K, kanamycin; TE, tetracycline; COT, co-trimoxazole; C, chloramphenicol.

The results of the antibiotic susceptibility patterns of the *E. coli* isolates were measured based on the zones of inhibition and interpreted as per CLSI guidelines (Supplementary Figure S4). The *E. coli* isolates showed different reactions to the antibiotics on Mueller Hinton agar; that is susceptibility, resistance or intermediate reaction. *E. coli* isolates showed the highest resistance to ampicilllin/cloxacillin (100%, *n* = 50) followed by tetracycline (62%, *n* = 31), erythromycin (44%, *n* = 22), co-trimoxazole (42%, *n* = 21), nalidixic acid (20%, *n* = 10), and kanamycin 12% (*n* = 6) with the least resistance observed against chloramphenicol (10%, *n* = 5) (Figure 3). There were significant differences (*p* ≤ 0.05) in the susceptibility profiles of kanamycin, chloramphenicol and nalidixic acid. There was no significant difference (χ^2^ = 7.3, *P* = 0.8372) in the frequency of antibiotic susceptibility patterns between the different sampling sites (Supplementary Figure S5).

#### 3.4.2 Multidrug resistance profile of *E. coli* isolates

**FIGURE 4:**
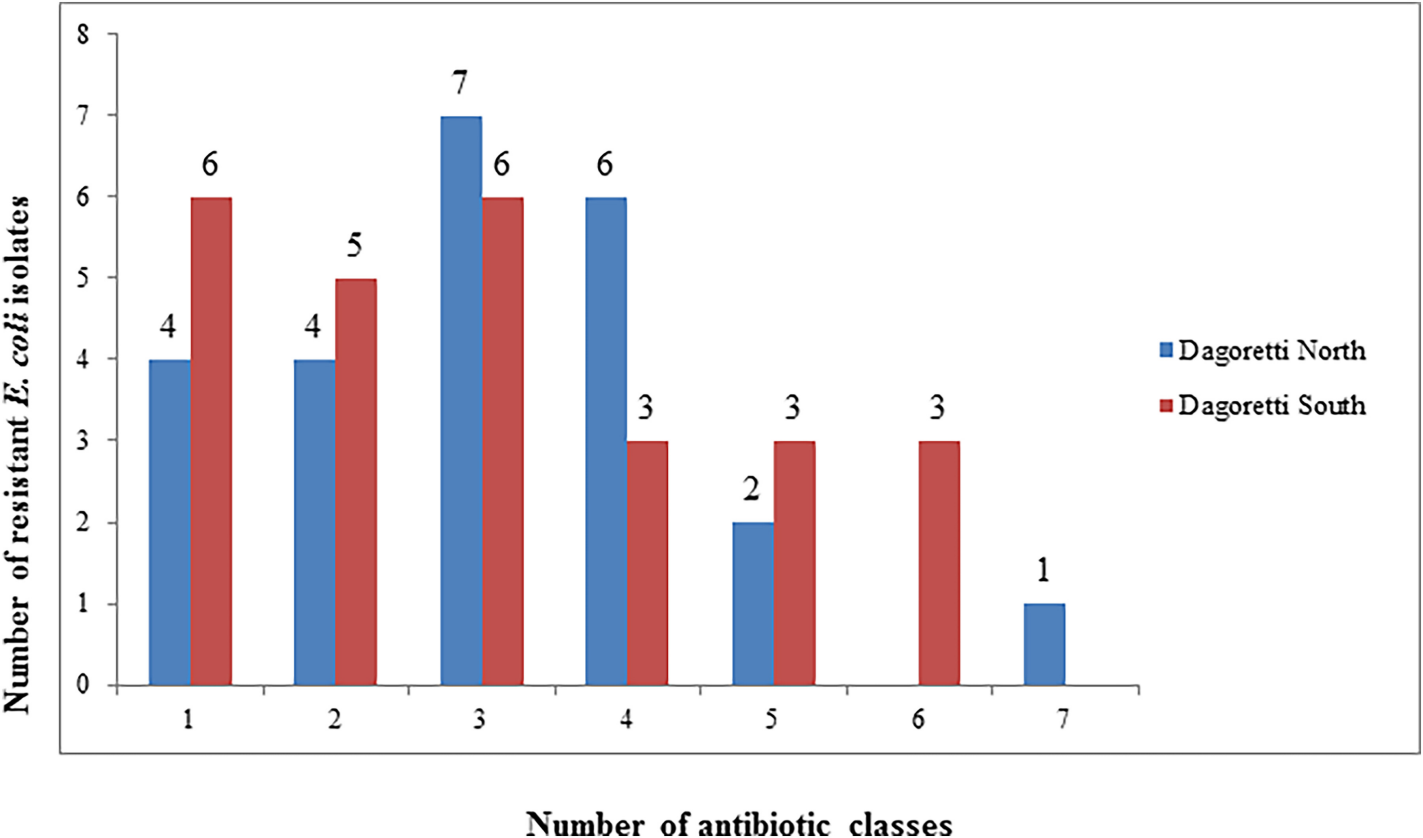
Antibiotic and multidrug-resistant profiles of *Escherichia coli* isolated from meat samples collected from different sampling sites in Dagoretti North and South sub-Counties. Multidrug-resistance profile of *E. coli* isolates, where the values 3–7 on X-asis indicate multidrug-resistant (MDR) isolates.

The isolates that exhibited resistance to three or more different classes of antibiotics were regarded as MDR. In the current study, all the 50 isolates of *E. coli* showed resistance to at least one antibiotic (Figure 4). Of the 50 *E. coli* isolates, 62% (*n* = 31) were MDR as they showed resistance to three or more antibiotic classes (Table 2). There was no significant difference (*P* = 0.83) in the multidrug resistance profiles of *E. coli* isolated from Dagoretti North and South sub counties. Multiple antibiotics resistance (MAR) index for the *E. coli* isolates ranged from 0.43 and 1.0 (Table 2).

**TABLE 2:** Antimicrobial resistance phenotype patterns and multiple antibiotic resistance (MAR) indices of *E. coli* (*n* = 50) and *Salmonella* spp. isolates from beef sold in Dagoretti North and South sub-Counties, Kenya.

Pathogen	Antimicrobial resistance pattern (antibiotics combination)	Number of antibiotic classes	Number of antibiotics	Number of isolates	Multiple antibiotic resistance (MAR) index
*Escherichia coli*	C_50_ + AX_10_ + TE_30_ + COT_25_ + E_15_ + NA_30_ + K_30_	7	7	1	1
	C_50_ + AX_10_ + TE_30_ + COT_25_ + E_15_ + NA_30_	6	6	2	0.86
	AX_10_ + TE_30_ + COT_25_ + E_15_ + NA_30_ + K_30_	6	6	1	0.86
	AX_10_ + TE_30_ + COT_25_ + E_15_ + NA_30_	5	5	2	0.71
	C_50_ + AX_10_ + TE_30_ + E_15_ + K_30_	5	5	1	0.71
	AX_10_ + TE_30_ + COT_25_ + E_15_ + K_30_	5	5	1	0.71
	C_50_ + AX_10_ + TE_30_ + COT_25_ + NA_30_	5	5	1	0.71
	AX_10_ + TE_30_ + COT_25_ + E_15_	4	4	7	0.57
	AX_10_ + TE_30_ + COT_25_ + NA_30_	4	4	2	0.57
	AX_10_ + TE_30_ + COT_25_	3	3	9	0.43
	AX_10_ + COT_25_ + E_15_	3	3	2	0.43
	AX_10_ + TE_30_ + E_15_	3	3	1	0.43
	AX_10_ + E_15_ + K_30_	3	3	1	0.43
*Salmonella* spp.	AX_10_ + E_15_ + K_30_ + TE_30_ + C_50_ + COT_25_ + NA_30_	7	7	2	1
	AX_10_ + E_15_ + TE_30_ + C_50_ + COT_25_ + NA_30_	6	6	2	0.86
	AX_10_ + E_15_ + K_30_ + TE_30_ + COT_25_	5	5	1	0.71
	AX_10_ + E_15_ + TE_30_ + COT_25_	4	4	1	0.57
	AX_10_ + E_15_ + K_30_ + TE_30_	4	4	1	0.57
	AX_10_ + E_15_ + TE_30_	3	3	3	0.43
	AX_10_ + TE_30_ + COT_25_	3	3	2	0.43

The antibiotic classes (and antibiotics) are Penicillin (Ampicillin/Cloxacillin [AX_10_, 10 μg]), Macrolide (Erythromycin [E_15_, 15 μg]), Quinoline (Nalidixic acid [NA_30_, 30 μg]), Aminoglycoside (Kanamycin [K_30_, 30 μg]), Tetracycline (Tetracycline [TE_30_, 30 μg]), Folate-pathway antagonist (Co-trimoxazole [COT_25_, 25 μg]) and Phenolic derivatives (Chloramphenicol [C_50_, 50 μg]).

#### 3.4.3 *Salmonella* spp. susceptibility patterns

**FIGURE 5:**
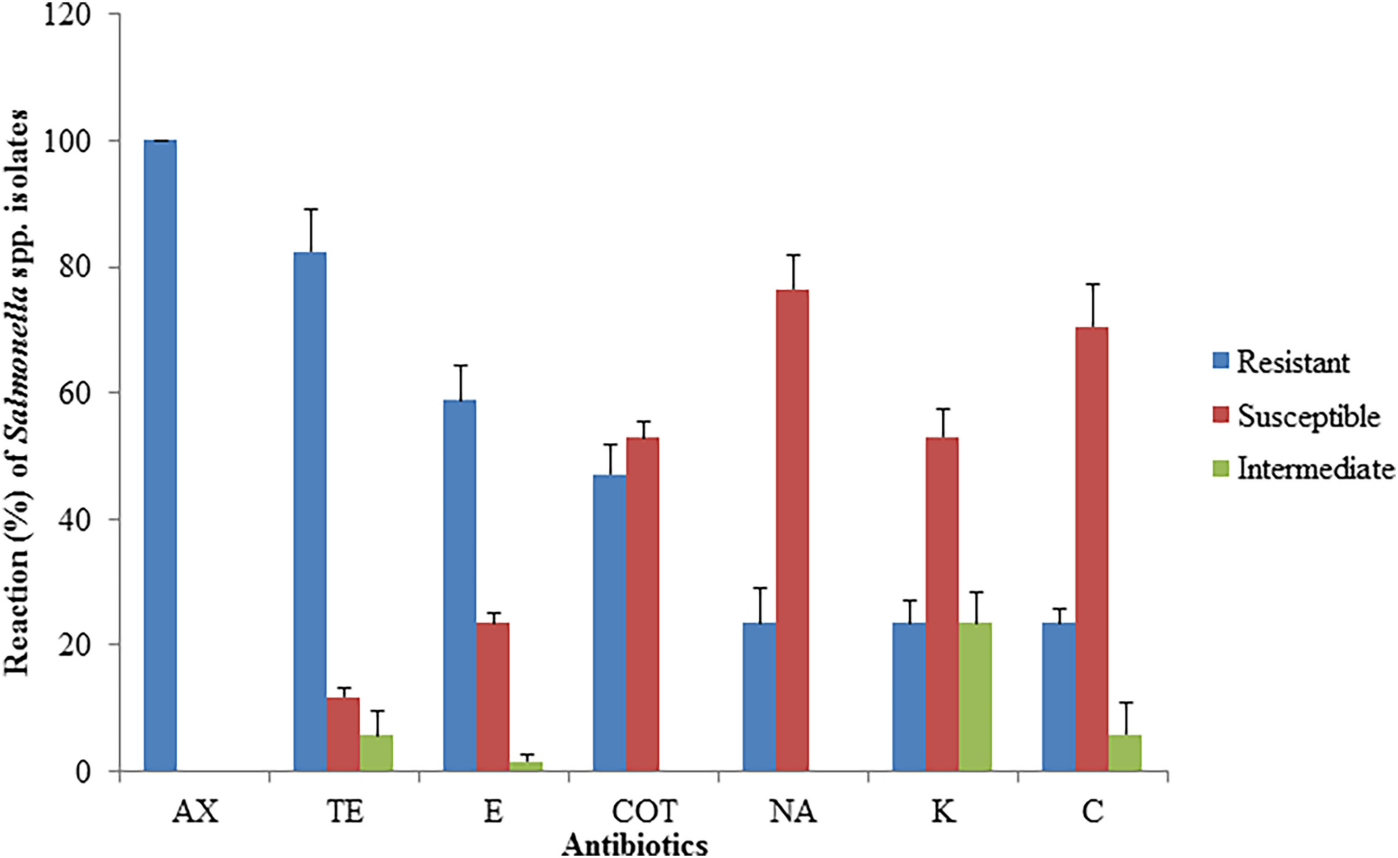
Antibiotic reactions of 17 *Salmonella* spp. isolates from different sampling sites in Dagoretti North and South sub counties to different antibiotics. The error bars represent standard error of the means. AX, ampicillin/Cloxacillin; E, erythromycin; NA, nalidixic acid; K, kanamycin; TE, tetracycline; COT, co-trimoxazole; C, chloramphenicol.

Seven antibiotics were used for testing the antibiotic susceptibility of *Salmonella* spp. isolates. Each of the isolates had varying profiles of antibiotic reaction (Supplementary Figure S6). Seventeen presumptive isolates of *Salmonella* spp. were randomly selected and subjected to the seven antibiotics and presented different susceptibility patterns. *Salmonella* spp. isolates showed the highest resistance to ampicilllin/cloxacillin (100%, *n* = 17) followed by tetracycline (82.4%, *n* = 14), erythromycin (58.8%, *n* = 10), co-trimoxazole (47.1%, *n* = 8), nalidixic acid (23.5%, *n* = 4), kanamycin (23.5%, *n* = 4) and chloramphenicol (23.5%, *n* = 4) (Figure 5). There were significant differences (*p* ≤ 0.05) in the susceptibility profiles of tetracycline, chloramphenicol and nalidixic acid. There was no significant difference (χ^2^ = 18.5, *P* = 0.1015) in the antibiotic susceptibility patterns of *Salmonella* spp. isolates from the different sampling sites (Supplementary Figure S7).

#### 3.4.4 Multidrug resistance profile of *Salmonella* spp. isolates

**FIGURE 6:**
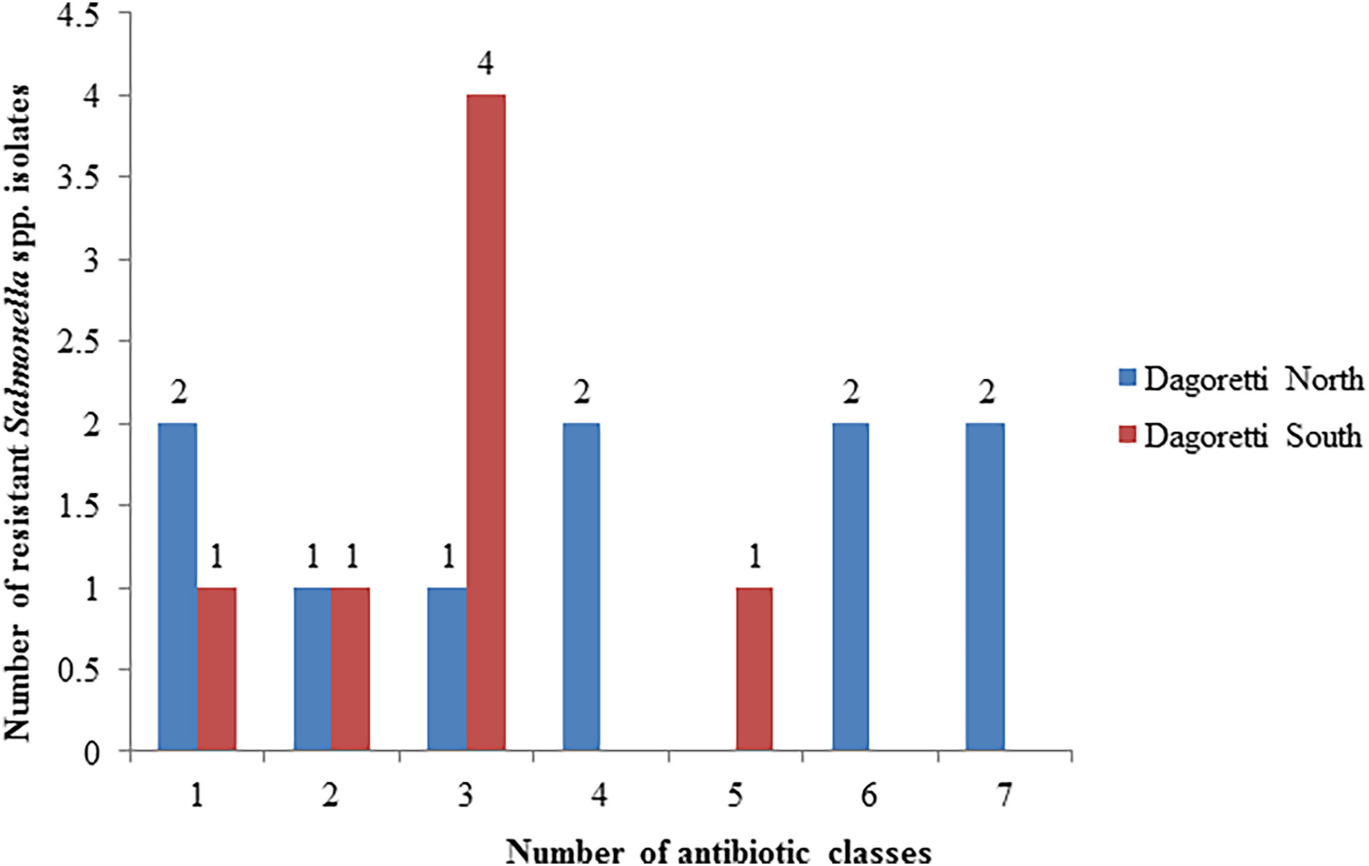
Antibiotic and multidrug-resistant profiles of *Salmonella* spp. isolated from meat samples collected from different sampling sites in Dagoretti North and South sub-Counties. Multidrug-resistance profile of *Salmonella* spp. isolates, where the values 3–7 on the X-asis indicate multidrug-resistant (MDR) isolates. The values 1, 2 and 4 presented above each of the bars represents the number of resistant *Salmonella* spp. isolates.

The multidrug resistance profiles of the *Salmonella* spp. isolates were analyzed. In the current study, all the 17 isolates of *Salmonella* spp. showed resistance to at least one antibiotic (Figure 6). Of the 17 *Salmonella* spp. isolates, 70.59% were multi-drug resistant showing resistance to three or more antibiotics (Table 2). Two isolates from Dagoretti North showed resistance to six antibiotics, and an additional two isolates from the same area were resistant to all seven antibiotics tested. In comparison, no isolates from Dagoretti South were resistant to either six or all seven antibiotics (Figure 6). Multiple antibiotics resistance (MAR) index for the *Salmonella* spp. isolates ranged from 0.43 and 1.0 (Table 2).

#### 3.4.5 Screening for antibiotic resistant genes

Ten *E. coli* and *Salmonella* spp. isolates were randomly selected based on results of antimicrobial resistance using the disc diffusion method for screening to determine the presence of antibiotic-resistant genes using specific primers. The *bla*_TEM_ and *bla*_CMY–2_ primers were used to detect genes associated with resistance to ampicillin/cloxacillin, while the *tet*A and *tet*C primers were used to screen for tetracycline resistance genes. The *sul* primer was used to identify genes associated with co-trimoxazole resistance, and the *cat*1 primer was used to detect genes encoding resistance to chloramphenicol.

**FIGURE 7:**
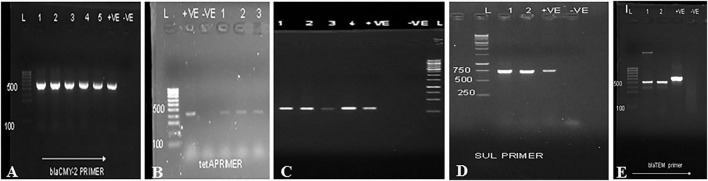
Agarose gel electrophoresis image showing PCR amplification of antibiotic genes. **(A)** *bla*_CMY–2_ of amplicons size 600 bp, samples are represented by 1 = A5, 2 = N10, 3 = C3, 4 = KK2 and 5 = N1. **(B)** *tet*A of amplicons size of 480 bp, samples are represented by 1 = N10, 2 = C3 and 3 = A2. **(C)** *cat*l of amplicons size 320 bp, samples are represented by 1 = KK2, 2 = A2 and 3 = B8. **(D)** *Sul* of amplicons size of 722 bp, samples are represented by 1 = A5, 2 = C3. **(E)** *bla*_TEM_ of amplicons size of 480 bp, samples are represented by 1 = N10, 2 = C3. L = GeneRuler 1 kb plus Ladder, + VE = Positive control and –VE = Negative control. Strains *E. coli* MAK-26 and *S. typhimurium* MAK-22 were used as positive controls for the PR reactions for detection of antibiotic resistance genes in MDR bacteria isolates (Mumbo et al., 2023).

Polymerase chain reaction was used to amplify the DNA, and the presence of amplified PCR products was confirmed by agarose gel electrophoresis. Ten samples were positive for *bla*_TEM_ and *bla*_CMY–2_ and amplified a specific PCR product of the expected size of 600 bp as observed on agarose gel electrophoresis (Figure 7). Two samples were positive for *cat*1 and amplified a specific PCR product of the expected size of 320 bp as observed on agarose gel electrophoresis (Figure 7), three samples were positive for *tet*A and *tet*C and amplified a specific PCR product of the expected size of 480 bp as observed on agarose gel electrophoresis (Figure 7) while five samples were positive for *Sul* and amplified a specific PCR product of the expected size of 722 bp (Figure 7). A summary for the amplifications of the resistant genes with the respective primers is presented in Table 3.

**TABLE 3:** Results showing the presence/absence of antibiotic-resistance genes in selected *E. coli* and *Salmonella* spp. Isolates.

Isolates	Sample ID	Antibiotic resistance genes					
		*blaTEM*	*blaCMY-2*	*tetC*	*tetA*	*Catl*	*sul*
*E. coli*	D1	+	+	−	−	−	−
	N10	+	+	+	+	−	−
	C3	+	+	+	+	−	−
	KK2	+	+	−	−	+	+
	N1	+	+	−	−	−	+
	A2	+	+	+	+	+	+
	B8	+	+	−	−	−	+
	N9	+	+	−	−	−	−
*Salmonella* spp.	A5	+	+	−	−	−−	+
	K4	+	+	−	−	−	−

(+) and (−) refers to presence and absence of antibiotic resistant gene, respectively.

### 3.5 Molecular identification and characterization of selected *E. coli* isolates

Molecular identification of the isolates was determined based on 16S rRNA amplification and sequencing. Positive results were indicated by the presence of the amplified bands of approximate size of 1,500 bp (Supplementary Figure S8). For all the 10 *E. coli* isolates analyzed, bands of amplified PCR products were observed at the appropriate size. The amplified PCR products were sequenced and the sequences compared with the reference sequences from the database.

The sequences of the amplified PCR products were subjected to basic local alignment search tool (BLAST) analysis. Based on BLAST search, four samples (N10, KK2, N9, and D1) were closely related and identified as *E. coli* with a similarity of between 99.19 and 99.51% while the rest of the samples (B8, C3, N1, and A2) were closely related and identified as *Enterobacter* species with similarity ranging from 99.53 to 99.82% (Supplementary Table S2). The sequences of 16S rRNA gene of eight MDR isolates were deposited in the NCBI database under accession numbers PV866776.1, PV866778.1, PV866780.1 and PV866781.1 for *E. coli*; PV866775.1 and PV866777.1 for *Enterobacter cloacae* and PV866779.1 and PV866782.1 for *Enterobacter hormaechei* (Supplementary Table S1).

**FIGURE 8:**
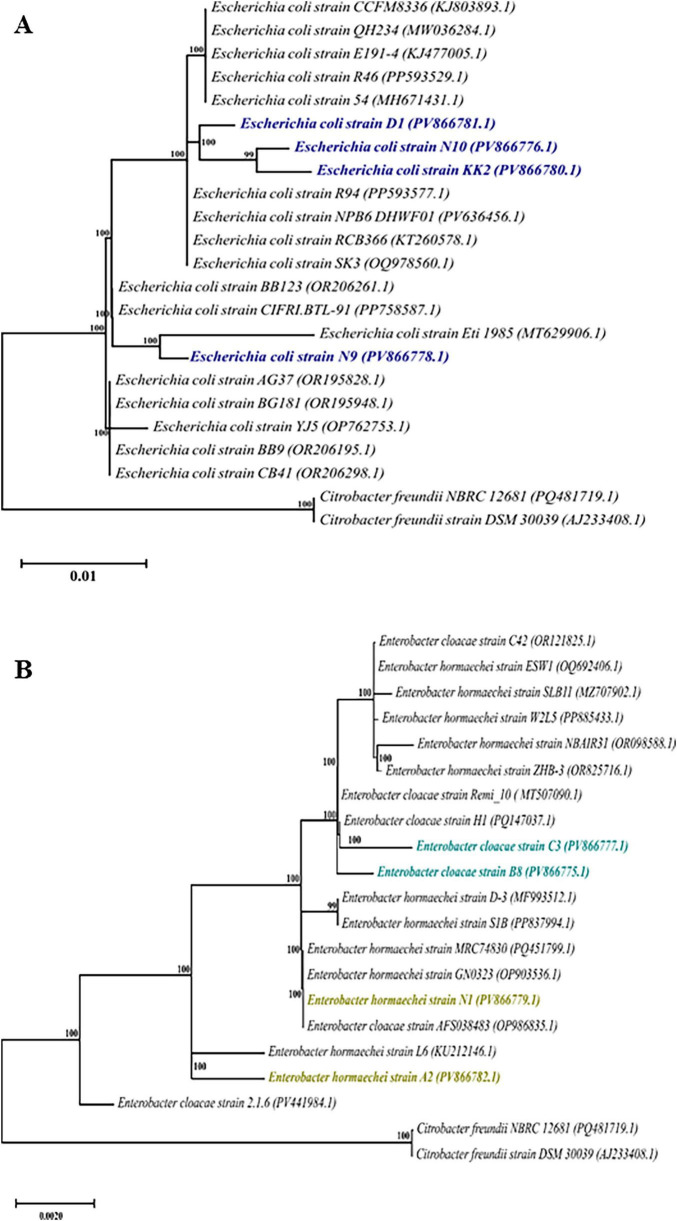
Maximum likelihood phylogenetic tree constructed using 16S rDNA gene sequences of *Escherichia coli* **(A)** and *Enterobacter* **(B)** isolates with reference sequences and *Citrobacter freundii* strains as the outgroup. *E. coli* sequences from this study are shown in navy blue. *Enterobacter* spp. Sequences from this study are shown in olive and teal colors. The tree with the highest log likelihood was selected, and bootstrap values (1,000 replicates) are indicated at the branch nodes. The Kimura 2-paramter model with a discrete Gamma distribution (+ G) was used to model the evolutionary rate variation among sites. The scale bar below the tree indicates the number of nucleotide base substitutions per site. The analysis was performed in MEGA11, with *Citrobacter freundii* as outgroup used in rooting the tree.

The phylogenetic analysis (Figure 8A) of 16S rRNA sequence of the four MDR *E. coli* isolates (PV866776.1, PV866778.1, PV866780.1, and PV866781.1) showed distinct clustering and had the same node showing that they both evolved from the same ancestor. The four isolates clustered together in the same cladograph and had 100% homology. The phylogenetic analysis (Figure 8B) of 16S rRNA sequences of the four MDR *Enterobacter* species isolates (PV866775.1 and PV866777.1 for *E. cloacae* as well as PV866779.1 and PV866782.1 for *E. hormaechei*) showed distinct clustering and had the same node showing that they both evolved from the same ancestor.

## 4 Discussion

The current study evaluated the prevalence and AMR profiles of *E. coli* and *Salmonella* spp. from raw beef from abattoirs and retail butcher shops in Dagoretti sub-County, Nairobi, Kenya. The findings showed the presence of *E. coli* and *Salmonella* spp. in raw beef. *Escherichia coli* were isolated from 69.8% of the beef samples collected. These findings compare with the reports of a similar study in Ghana where 86.7% of the beef samples were positive for *E. coli* (Adzitey et al., 2020). However, other studies reported lower contamination of beef samples: 54% in Dharan (Bantawa et al., 2018), 35.2% in Central Ethiopia (Bersisa et al., 2019), 43.8% in Addis Ababa, Ethiopia (Zerabruk et al., 2019) and 32.5% in Eastern Java province, Indonesia (Soepranianondo et al., 2019). The observed differences in prevalence could be due to the differences in hygiene handling practices during processing and transportation. The high contamination levels in this study could be attributed to non-adherence to general principles of food hygiene and Hazard Analysis Critical Control Point principles during slaughtering, transportation, storage and sale of beef meat. *Salmonella* spp. was isolated in 5.7% of the samples analyzed. These findings compare with a similar study done in Abuja, Nigeria where *Salmonella* spp. was recovered in 1.5% of the beef samples (Bawa et al., 2020). Similarly, a study in Eastern Java province, Indonesia reported a detection rate of 2.5% (Soepranianondo et al., 2019). The detection of *Salmonella* spp. in this study is lower than in other studies where *Salmonella* spp. contamination was reported at 34% in Dharan, Eastern Nepal (Bantawa et al., 2018), 9.9% in Bishoftu, Central Ethiopia (Bersisa et al., 2019), and 29.17% in Addis Ababa, Ethiopia (Zerabruk et al., 2019). As set out in Kenyan Standard KS 317-1 Carcasses and meat cuts for bovine meat-Specification Part 1: Beef and veal, *Salmonella* spp. should not be detected in beef. *Salmonella* spp. is a microorganism of public health concern, and therefore its detection in this study is an issue of concern based on its pathogenicity and potential to cause adverse illness and even death.

Antimicrobial resistance has become a major issue of public health concern. In this study, *E. coli* isolates were subjected to seven antibiotics commonly used in human and animal treatment. It was observed that 80% of the isolates were resistant to three or more antibiotics, while 20% of the isolates were resistant to one antibiotic. The multidrug resistance reported in this study concurs with the 82.5% multidrug resistance of a similar study carried out in Northwest Spain (González-Gutiérrez et al., 2020). In Kenya, multidrug resistant *E. coli* was reported to be 30% in 2001 (Mitema et al., 2001). However, a recent study indicated that multidrug resistance has increased to 81.5% (Deng et al., 2024), which is similar to the findings in the current study. This shows that resistance to commonly used antibiotics is high and rising. It is of concern to note that all the *E. coli* isolates (100%) were found to be resistant to Ampicillin/Cloxacillin. A similar study in Nigeria showed the same results where 100% of the *E. coli* isolates were resistant to Ampicillin (Datok et al., 2021). Ampicillin is an extended penicillin that is effective against both gram negative and gram-positive bacteria. It is used to treat a wide range of infections including urinary tract infections, enteric fever, respiratory tract infection among others (Maqbool et al., 2020).

Resistance to tetracycline, which is a commonly used antibiotic, was 62%. These findings compare with the report of a study in Northwest Spain where 55% of the *E. coli* isolates were resistant to tetracycline. Similarly, 60.3% of *E. coli* isolates from chicken in Kenya were resistant to tetracycline. In a study done in Ghana, *E. coli* isolates from meat had a higher (73.3%) resistance to tetracycline (Adzitey et al., 2020). More than half (58%) of the *E. coli* isolates in the current study were resistant to co-trimoxazole. A similar study in Nigeria reported that 77% of the *E. coli* isolates were resistant to co-trimoxazole (Datok et al., 2021). In this study, resistance to Erythromycin was 44%, these findings are lower than a similar study in Ghana where *E. coli* isolates showed 85% resistance to Erythromycin (Adzitey et al., 2020). Resistance to nalidixic acid, kanamycin and chloramphenicol were slightly lower at 20, 12, and 10%, respectively.

The findings for antibiotic resistance in this study were supported by the detection of antibiotic resistant genes in the *E. coli* isolates. The *bla*_TEM_ and *bla*_CMY–2_ genes associated with resistance to ampicillin/cloxacillin were detected on 100% of the screened samples, while 30% of the isolates were positive for *tet*A and *tet*C genes associated with tetracycline resistance. The *sul* gene associated with co-trimoxazole resistance was present in 50% of the *E. coli* isolates while 20% of the isolates were positive for cat1 primer used to detect the gene encoding resistance to chloramphenicol. *Salmonella* spp. isolates were also subjected to antibiotics susceptibility/resistance tests using seven commonly used antibiotics. Eighty two percent of the isolates were resistant to two or more antibiotics while 18% were resistant to one or two antibiotics. The multidrug resistance in this study is higher than that of a similar study done in Jimma, Southwest Ethiopia where the multidrug resistance was 58.3% (Geresu and Desta, 2021). All the *Salmonella* spp. isolates were resistant to Ampicillin/Cloxacillin, while 82% were resistant to tetracycline. A similar study in Abuja, Nigeria reported 100% resistance of *Salmonella* spp. isolates to both ampicillin and tetracycline (Bawa et al., 2020). The resistance patterns for *E. coli* and *Salmonella* spp. isolates to antibiotics in this study is worrying. The antibiotics used in this study are categorized as either critically important or highly important according to WHO list of critically important antimicrobials for human medicine. Ampicillin/cloxacillin, erythromycin, kanamycin and nalidixic acid are classified as critically important while chloramphenicol, tetracycline and co-trimoxazole (trimethoprim and sulfamethoxazole) are classified as highly important (World Health Organization [WHO], 2018).

The level of resistance in this study can be attributed to misuse/overuse of antibiotics in animal production. In a study conducted to determine the use of antibiotics by poultry farmers in Kiambu County, Kenya, it was reported that farmers used antibiotics for prophylactics and to increase egg production. The commonly used antibiotics in poultry production include tetracycline, Macrolides (erythromycin), aminoglycosides, polymyxin, sulfonamides and penicillin (Ampiclox-ampicillin/cloxacillin) (Kariuki et al., 2023). In another study carried out among livestock farmers in Western Kenya, it was observed that 40% of veterinary antimicrobials were sold without prescription and that there was little knowledge on the dangers associated with antimicrobial resistance (AMR) and antimicrobial use (AMU) (Kemp et al., 2021).

In the current study, all the MDR *E. coli* isolates from raw beef were successfully amplified using 16S rRNA gene. Basic local alignment search tool (BLAST) and phylogenetic analysis of 16S rRNA gene sequences of the MDR *E. coli* isolates was done and isolates N10, D1, N9 and KK2 were identified as *E. coli*. Isolate B8 and C3 were classified as *Enterobacter cloacae* by both BLAST and phylogenetic analysis. Phylogenetic trees are reliable because they are known to show consistency of the relationship between organisms (Anggraini et al., 2018). Isolate A2 and N1 was identified as *Enterobacter hormaechei* using BLAST and phylogenetic analysis. Isolation of *Enterobacter cloacae* in this study is a major public health concern because of its potential in antimicrobial resistance. The phylogenetic tree showed that the four MDR *E. coli* isolates may have genetic relationship with reference strains from the databases. Based on the phylogenetic tree, each species formed a monophyletic clade indicating that the isolates were from the same ancestral lineage. This indicates that the bacteria isolated from raw beef from the study area had close phylogenetic relationships. The same findings have also been reported by previous studies by Abuelhassan et al. (2016) who explained in their phylogenetic tree that the studied *E. coli* isolates were in a clade close to and similar to those obtained in other parts of the world. The study showed that all *E. coli* isolates were similar to *E. coli* strains isolated in other countries which were pathogenic *E. coli*. The presence of pathogenic and MDR *E. coli* in beef indicates the potential for zoonosis.

Our study has notable limitations. The limited geographic coverage may not adequately reflect the broader informal retail market landscape in Kenya, thereby affecting the generalizability of the findings. The sample size of 10 randomly selected isolates for resistance gene screening remains limited and may not adequately capture the full spectrum of resistance or variety of mechanisms – such as diverse-lactamases, efflux pumps, or target site mutations present across the entire collection. Furthermore, rare and/or emerging résistance genes present in the entire sample collection but not in the randomly selected 10 isolates may be missed. Future studies should include larger sample size for resistance gene screening to uncover and characterize novel and previously unrecognized resistance mechanisms. In addition, molecular typing of *Salmonella* spp. was not performed to confirm the specific phylogroup *Salmonella* spp. Identifying specific phylogroup *Salmonella* spp. is essential for developing control strategies to reduce *Salmonella* spp. infections in the human population. The absence of whole-genome sequencing (WGS) may have resulted in overlooking critical resistance genes. To gain a more comprehensive understanding of antimicrobial resistance and critical resistance genes, future research should perform a genomic characterization of MDR *E. coli* and *Salmonella* spp. isolates using WGS, to gain insight into the diversity of pathotypes, phylogroups, serotypes, and plasmid replicons, as well as the distribution of the virulence and antimicrobial resistant genes.

## 5 Conclusion

The findings from the current study demonstrated that beef from abattoirs and retail butcher shops in Dagoretti sub-County, Nairobi, Kenya is highly contaminated with *E. coli* and with *Salmonella* spp. Although the cause of the high levels of *E. coli* contamination was not investigated, the contamination can be attributed to non-adherence to general principles of food hygiene and poor handling of meat during slaughter, transportation, storage and retail. *Escherichia coli* and *Salmonella* spp. isolates from beef had high antimicrobial resistance to commonly used antibiotics in humans and animals. There are antibiotics that have been classified by the WHO as either critical or highly important. All the screened *E. coli* isolates had at least two antimicrobial resistant genes, all the isolates were positive for *bla*_TEM_ and *bla*_CMY2_ genes. Antimicrobial resistant genes have the potential of being transferred from one microorganism to the other including pathogenic and non-pathogenic. This event is likely to contribute to increased incidences of antimicrobial resistance. The molecular characterization of *E. coli* using 16S rRNA and subsequent phylogenetic analysis indicates an evolutionary relationship of the *E. coli* isolates with other pathogenic *E. coli* strains from other countries. This indicates the potential risk of zoonosis. Further studies are recommended to investigate the occurrence of *E. hormaechei* and *E. cloacae* in beef in Kenya as they are significant microorganisms in colistin resistance.

## Data Availability

The datasets presented in this study can be found in online repositories. The names of the repository/repositories and accession number(s) can be found in the article/Supplementary material.

## Footnotes

1
https://www.addgene.org/protocols/dna-quantification/

2
https://blast.ncbi.nlm.nih.gov/Blast.cgi

3
https://submit.ncbi.nlm.nih.gov/about/genbank/
