# Supplementary material for: Occurrence and antimicrobial susceptibility patterns of Escherichia coli and Salmonella species in raw beef from abattoir and retailer shops in Nairobi, Kenya
Source: Front Microbiol. 2026 Jun 15;17:1808597. doi: 10.3389/fmicb.2026.1808597 (PMC13310884; doi:10.3389/fmicb.2026.1808597)
Supplement: Supplementary file 1 [file Table_1.docx]

Supplementary Material

**Occurrence and antimicrobial susceptibility patterns of *Escherichia coli* and *Salmonella* species in raw beef from abattoir and retailer shops in Nairobi, Kenya**

**Caroline C. Kasiwai^1,2^, Joshua M. Njiru^3*^, Maina J. Wagacha^1^ and Evans N. Nyaboga^4*^**

^1^Department of Biology, University of Nairobi, Nairobi, Kenya

^2^Testing Department, Kenya Bureau of Standards, Nairobi, Kenya

^3^Research and Development Department, Kenya Bureau of Standards, Nairobi, Kenya

^4^Department of Biochemistry, University of Nairobi, Nairobi, Kenya

*** Correspondence:** [nyaboga@uonbi.ac.ke](mailto:nyaboga@uonbi.ac.ke) (E.N.N) and [njiruj@kebs.org](mailto:njiruj@kebs.org) (J.M.N)

**Supplementary Tables**

**Supplementary Table S1** Primer sequences for detection of resistant genes in antibiotic resistant bacteria and their respective amplicons size

| Target gene | Primer | Sequence of primers | Amplicons size (bp) |
| --- | --- | --- | --- |
| *Sul*2 | Sul2-F | CGGCATCGTCAACATAACC | 722 |
|  | Sul2-R | GTGTGCGGATGAAGTCAG |  |
| *tet*A | tetA-F | GCTACATCCTGCTTGCCTTC | 280 |
|  | tetA-R | CATAGATCGCCGTGAAGAGG |  |
| *tet*C | tetC-F | CTTGAGAGCCTTCAACCCAG | 480 |
|  | tetC-R | ATGGTCGTCATCTACCTGCC |  |
| *Cat*1 | catl-F | AGTTGCTCAATGTACCTATAACC | 320 |
|  | catl-R | TTGTAATTCATTAAGCATTCTGCC |  |
| *bla*_TEM_ | blaTEM-F | TTGGGTGCACGAGTGGGT | 500 |
|  | blaTEM-R | TAATTGTTGCCGGGAAGC |  |
| *bla*_CMY-2_ | blaCMY-2-F | ATAACCACCCAGTCACGC | 600 |
|  | blaCMY-2-R | CAGTAGCGAGACTGCGCA |  |

**Supplementary Table S2** BLAST analysis of 16S rRNA gene sequences of *Escherichia coli* isolated from beef collected from Dagoretti North and South sub-Counties.

| **Sample ID** | **No. of bp** | **Coverage (%)** | **E-value** | **Percentage identity** | **Closest match from BLAST search** | **Accession Numbers of the isolates** |
| --- | --- | --- | --- | --- | --- | --- |
| B8 | 1113 | 100 | 0.0 | 99.82 | *Enterobacter cloacae* | PV866775.1 |
| N10 | 1413 | 100 | 0.0 | 99.29 | *Escherichia coli* | PV866776.1 |
| C3 | 1114 | 100 | 0.0 | 99.73 | *Enterobacter cloacae* | PV866777.1 |
| N9 | 1220 | 100 | 0.0 | 99.43 | *Escherichia coli* | PV866778.1 |
| N1 | 1309 | 100 | 0.0 | 99.85 | *Enterobacter hormaechei* | PV866779.1 |
| KK2 | 1355 | 100 | 0.0 | 99.19 | *Escherichia coli* | PV866780.1 |
| D1 | 1423 | 100 | 0.0 | 99.51 | *Escherichia coli* | PV866781.1 |
| A2 | 1480 | 100 | 0.0 | 99.53 | *Enterobacter hormaechei* | PV866782.1 |

## Supplementary Figures

**Supplementary Figure S1** Map of Kenya showing Dagoretti South and North sub-Counties. (Source: IEBC March 2012).


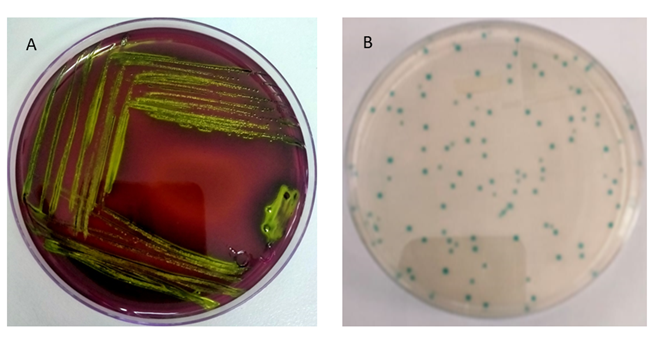


**Supplementary Figure S2** Colony characteristics of *E. coli* isolated from beef meat samples on Eosin Methylene blue Levine agar (A) and on TBX agar (B)


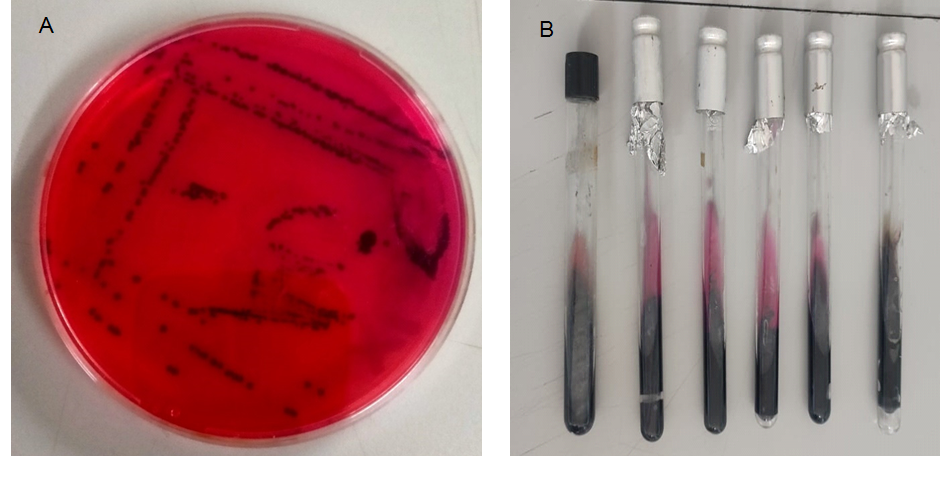


**Supplementary Figure S3** Characteristic colonies of *Salmonella* on xylose lysine decaboxylase medium, XLD (A) and biochemical reaction on tripple sugar iron agar, TSI (B)

**
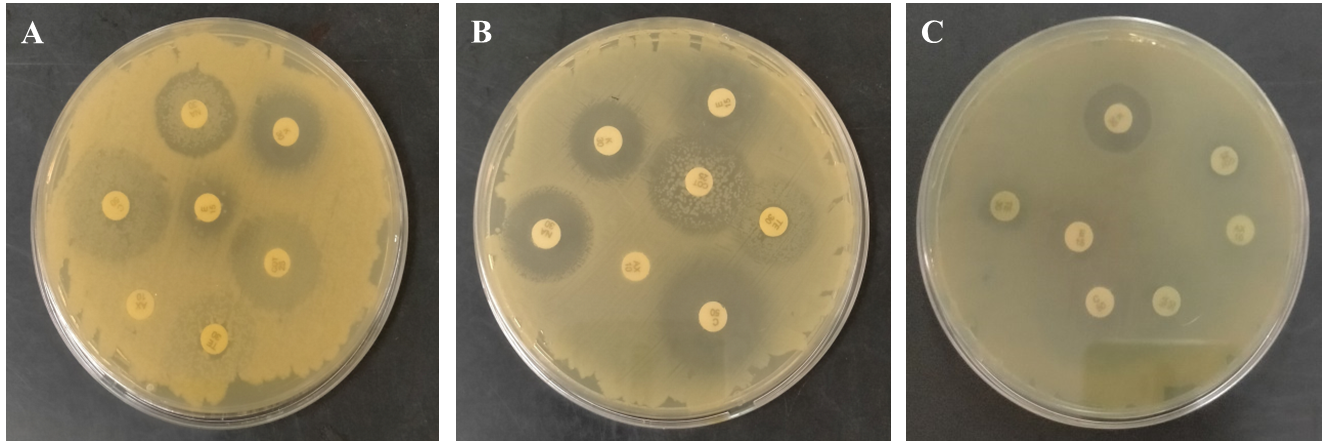
**

**Supplementary Figure S4** Antibiotic susceptibility/resistance testing of *E. coli* isolates obtained from beef samples collected from different sites in Dagoretti North and South sub-Counties using seven antibiotics on Mueller Hinton Agar. A, B and C represent examples of the zones of inhibition measured.

**Supplementary Figure S5** Susceptibility profiles of *E. coli* isolates from 50 beef samples collected from different sampling sites in Dagoretti North and South sub-Counties.

The error bars represent standard error of the means.


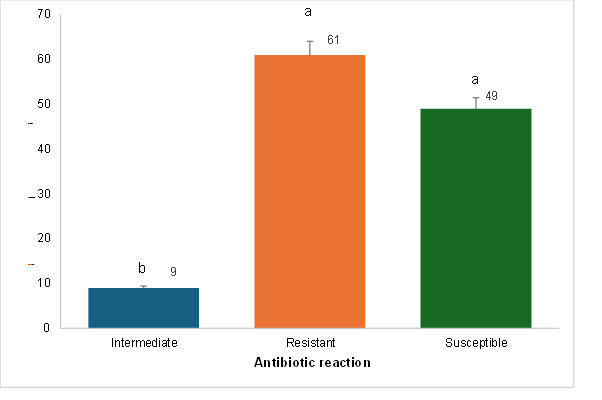


**Supplementary Figure S6** Antibiotic reaction of *Salmonella* isolated from meat samples collected from different sampling sites in Dagoretti North and South sub-Counties. Values are means ± standard errors of the means. Bars having similar lower case letters are not significantly different at p > 0.05.

**Supplementary Figure S7** The susceptibility profiles of 23 *Salmonella* spp. isolated from beef meat collected from different sampling sites in Dagoretti North and South sub-Counties.

The error bars represent standard error of the means.


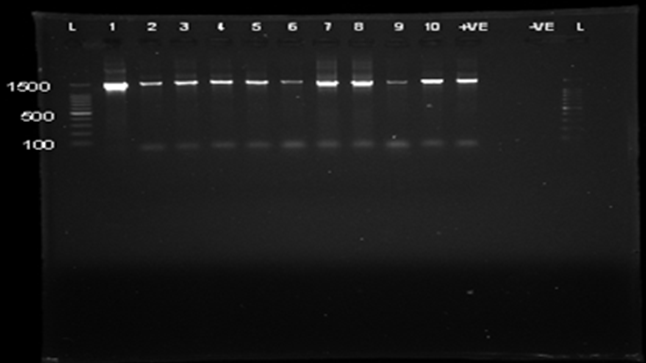


**Supplementary Figure S8** Agarose gel electrophoresis image showing PCR amplification of 16S rRNA of *E. coli* isolates. Approximately 1500 bp for each of the 10 *E. coli* isolates was amplified where L = GeneRuler Ladder 1 = N9, 2 = kk2, 3 = D1, 4 = B8, 5 = N10, 6 = C3, 7 = A2, 8 = N1, 9 = A5, 10 = K4 +VE = Positive control and –VE = Negative control.
